# Experiences From an Internet-Delivered Treatment Program for Individuals With Obesity: Pilot Study

**DOI:** 10.2196/79853

**Published:** 2026-04-17

**Authors:** Agneta Anderzen-Carlsson, Annika Imhagen, Stefan Jansson, Marije Galavazi, Jan Karlsson

**Affiliations:** 1 University Health Care Research Centre, Faculty of Medicine and Health, Örebro University, Örebro University Hospital, S-house, Örebro, 70185, Sweden, 46 196025846; 2 Department of Internal Medicine, Örebro University, Örebro, Sweden

**Keywords:** cognitive behavioral therapy, internet-based intervention, multimethods, obesity, pilot projects, social stigma

## Abstract

**Background:**

The prevalence of obesity is a global health challenge, as obesity is associated with various comorbidities, reduced quality of life, and increased mortality. Providing effective treatment to improve health and quality of life for people with obesity is a major health care concern. Internet-delivered treatment (IDT) is an alternative treatment that increases patient accessibility and reachability; however, pilot testing is required before such interventions are evaluated in full-scale studies or implemented.

**Objective:**

This study aims to investigate the feasibility and user-friendliness of an IDT program for obesity (IDT-O); to evaluate body weight, dietary habits, physical activity, psychosocial functioning, and experiences of treatment in those who completed the 6-month treatment; and to investigate the dropouts’ experiences of the treatment.

**Methods:**

A prospective 1-year observational approach, evaluated through a multimethod research design, was adopted. Inclusion criteria were age 18 years and older, BMI of ≥30 kg/m^2^, or BMI of 28‐29.9 kg/m^2^ with obesity-related comorbidity. Participants were offered a 6-month therapist-assisted IDT-O program providing evidence-based obesity treatment, behavioral and lifestyle support, and strategies to address weight stigma. BMI, participants’ dietary habits, self-reported physical activity, psychosocial functioning, experiences of treatment effects, and treatment satisfaction were measured before treatment and after 6 and 12 months. Dropouts were followed up through qualitative interviews.

**Results:**

A total of 20 participants (17 females and 3 male; mean age 44.2, SD 16.4 years) started the IDT-O program, and 35% (7/20) completed all 12 modules. Ten (8 females) out of 13 dropouts were interviewed. Both quantitative and qualitative findings showed that participants were generally satisfied with the content and design of the intervention. Those who completed the IDT-O lost some weight (mean 2.0%, 95% CI −1.09 to 5.13), reported improved dietary habits (effect size [ES] 0.25, 95% CI −0.51 to 1.00), increased physical activity (ES 0.93, 95% CI −0.08 to 1.87), and improved psychosocial functioning (distress: ES 0.43, 95% CI 0.‐0.37 to 1.19; avoidance: ES 0.67, 95% CI −0.18 to 1.48), 6 months after completing the treatment. The qualitative analysis of the interviews revealed “The programme was OK, but it does not suit everyone” as the main theme. The main themes were based on the 3 subthemes: “It wasn’t for me,” “There were good things,” and “There are things to improve.”

**Conclusions:**

The findings indicate that the IDT-O holds potential as a treatment for people with obesity, although one limitation is that only 35% (7/20) of the participants completed the pilot program. Improvements in lifestyle habits and psychosocial functioning were observed in those who completed the IDT-O, but these findings are preliminary and need to be confirmed in a more comprehensive study. The issue of nonadherence underscores the importance of both thoroughly assessing patients before treatment and further development of IDT-O programs.

## Introduction

The prevalence of obesity has increased over the past 30 years and represents a global health challenge [1]. Obesity is a chronic disease associated with various comorbidities, reduced quality of life (QoL), and increased mortality risk [2]. Providing effective treatment to improve health and QoL for the large proportion of the population with obesity is a major health care concern.

Lifestyle interventions focusing on dietary habits and physical activity are central components of weight loss treatment [3], but in-person treatment is staff-intensive and entails high costs, limiting the number of people who can receive optimal care. Internet-delivered treatment (IDT) is an alternative that increases accessibility and reachability for the patient [4] and is cost-efficient [5,6]. Through the use of cognitive-behavioral therapy (CBT) techniques, treatment via the internet provides therapists with good opportunities to guide patients and provide support for behavioral and lifestyle changes [7], although they face several professional challenges when moving from treatment in person to IDT [8,9]. A review of studies comparing therapist-assisted CBT via the internet (iCBT) versus face-to-face interventions for the treatment of psychiatric and somatic disorders found similar outcome effects [10].

Several reviews have demonstrated that IDT programs for the treatment of obesity can have beneficial effects in terms of behavioral and lifestyle changes, as well as weight reduction and health improvements [11-13]. However, the designs of different program packages vary greatly in terms of mode of delivery, the behavioral techniques and components included, unguided or therapist-assisted support, and the intensity and duration of treatment, making it difficult to determine which elements of the programs actually produce behavioral change. The outcomes in terms of weight loss and health improvements vary, and each IDT program should therefore be evaluated separately to ensure its effectiveness. Studying the experiences of both completers and dropouts is essential when evaluating IDTs. By incorporating feedback from noncompleters, valuable insights can be gained to improve program design and treatment strategies.

A CBT-inspired, 6-month IDT program for individuals with obesity (IDT-O) was developed at the obesity unit at Örebro University Hospital in Central Sweden. The IDT-O program was based on an in-person group treatment program that has been routinely used to treat people with obesity since 2010 [14]. The goals of the IDT-O intervention were primarily to offer evidence-based knowledge about obesity, to provide support for behavioral and lifestyle changes that can contribute to reduced body weight and improved health status, and to mitigate the negative effects of weight stigma. Evaluation of the program is necessary before implementing the program in clinical practice.

The aims of this pilot study were to investigate the feasibility and user-friendliness of the IDT-O program; to evaluate treatment outcomes regarding body weight, dietary habits, physical activity, and psychosocial functioning in those who completed the 6-month treatment; and to investigate dropouts’ experiences of the treatment.

## Methods

### Study Design

The pilot study was a prospective 1-year observational study, evaluated via a multimethod research design [15]. The participants answered questionnaires, height and weight were measured, and interviews with dropouts were conducted.

### Participants and Setting

Patients referred for assessment and treatment at the obesity unit at the Örebro University Hospital, which is located in an urban area of Central Sweden, were invited to participate. Those who wanted to participate in the program were consecutively included. Referrals to the obesity unit of patients with obesity can be made by health care professionals, such as general practitioners or public health nurses. At the obesity unit, psychologists, dietitians, physiotherapists, and physicians are employed. In this study, the intervention was delivered by all staff, except the physician, and the providers are referred to as therapists throughout the paper. The study ran from November 2019 to January 2021.

The inclusion criteria were patients aged 18 years or older and either a BMI of ≥30 kg/m^2^ or a BMI of 28‐29.9 kg/m^2^ along with concomitant hypertension, prediabetes, type 2 diabetes, coronary artery disease, hyperlipidemia, hepatic steatosis, sleep apnea, or polycystic ovary syndrome. The exclusion criteria were severe mental illness, an ongoing eating disorder, pregnancy, breastfeeding, cancer in active treatment, weight loss treatment in the last 6 months, active abuse of alcohol or drugs, and being unable to express oneself in Swedish in speech and writing.

As part of the study, 3 individual visits to the obesity unit were included, before participation in the IDT-O, after completion of treatment at 6 months, and at the 12-month follow-up. The IDT-O started within 2 weeks of the first visit. Dropouts were interviewed after they had terminated their participation.

### IDT for Obesity

Participants were provided with access to the IDT-O program via a secure login to the Swedish national health e-services called 1177.se. This platform is used for digital treatment and for digitally based contact with the official health care system in Sweden. The program lasted for 6 months and comprised 12 treatment modules with participants working for 2 weeks per module. The main content of the modules is shown in Table 1. The modules primarily consisted of text, but videos and images were also included. Each module ended with 1 or more practice tasks to be completed before the next module was started. Each participant had regular written contact with 1 of 4 therapists at the obesity unit via the treatment platform; the same therapist provided written feedback on completed practice tasks and answered questions from the participant.

**Table 1.** Main content of the modules of the IDT-O^a^.

Module	Main content	Task
1. Introduction	Overview of the program structure, what to expect, and goal-setting introduction to the logbook	Completion of the “My Goal, My Plan” questionnaire
2. Cornerstones of healthy lifestyle habits and the disease obesity	Healthy lifestyle behaviors; causes and consequences of obesity	Recording a 4-day food diary
3. Goal setting	Introduction to functional goal setting using the SMART model^b^; weight versus health	Formulation of personal goals within the ‘My Goal, My Plan’ framework
4. Dietary choices	Information on nutrition, including meal rhythm and portion sizes	Recording of meal rhythm and daily vegetable intake
5. The change process	Emphasis on self-monitoring as the initial step in behavior change, overview of stages in the change process, and identification of potential risk situations	Identification and management of risk situations
6. Physical activity	Reduction of sedentary behavior, general recommendations for physical activity and the physiological benefits of regular exercise, and common barriers to physical activity	Daily step count recording and completion of an activity log
7. Life balance	Exploration of life balance, including stress physiology, the impact of stress on health and the role of sleep; stop and reflect	Engagement in a mindfulness exercise (“Breathe”) for a duration of 1 week
8. Eating behavior	Examination of unconscious eating patterns, emotional eating, and the physiological mechanisms of hunger and satiety; strategies for managing food cravings	Completion of a food diary (based on hunger-satiety patterns); situational analysis related to emotional eating triggers
9. Problem-solving	Introduction to problem-solving in 6 steps	Application of the 6-step problem-solving model to real-life scenarios
10. Societal body ideals and norms	Exploration of societal body image ideals, the internalization of negative body thoughts, and the role of body activism; emphasis on self-compassion	Identifying characteristics of admired individuals and recognizing positive aspects of one’s own body
11. Success factors	Success factors in weight loss; introduction to strategies for weight maintenance	Completion of a personalized maintenance plan utilizing the “Traffic Light”^c^ model
12. This is my life	Review of the program and reinforcement of personal autonomy in the treatment process	Self-monitoring of healthy lifestyle behaviors (eg, food diary, meal timing, vegetable intake, and physical activity); comparison of current progress with earlier records

a Treatment lasts for 6 months. Participants work with each module for 2 weeks, completing tasks after each module on which they receive written feedback from the therapist.

b The SMART (Specific, Measurable, Achievable, Relevant, and Time-bound) model: A method for setting goals.

c “The traffic light”: strategies to maintain healthy lifestyle habits and a stable weight. Green light: I am on the right track. Yellow light: I am moving away from my goals. Red light: I have lost focus.

### Outcome Measures

#### Overview

Measurements were taken during visits to the obesity unit before treatment and after 6 and 12 months, unless otherwise noted. Body weight— with participants wearing light clothing but no shoes—was measured to the nearest 0.1 kg using an electronic scale. Height was measured to the nearest 0.01 m at baseline, with participants in a standing position and without shoes. BMI was calculated. The questionnaires were answered in paper by the participants on site and thereafter handed over to the therapist.

#### Dietary Habits

Dietary habits were assessed using the Swedish National Board of Health and Welfare’s 4 validated dietary index questions [16]. These questions measure the frequency of consumption of (1) vegetables and root vegetables, (2) fruits and berries, (3) fish and shellfish, and (4) rolls, sweets and chocolates, and sugar-sweetened drinks. More frequent consumption gives higher scores on the first 3 questions, while the opposite scoring is used for the fourth question. The responses are summed to a total score ranging from 0 to 12, where a higher score signifies more favorable dietary habits.

#### Self-Reported Physical Activity

Self-reported physical activity was assessed using the Swedish National Board of Health and Welfare’s 2 validated questions about the frequency of vigorous and moderate physical activity [17]. The 2 questions are answered on a 6-point response scale (0, <30, 30‐60, >60‐90, >90‐120, or >120 minutes per week), where 0 minute per week is coded as 1 and >120 minutes per week as 6. The response for moderate activity is scored from 1 to 6, whereas the response for vigorous activity is multiplied by 2 to account for a higher intensity. A summary score is then calculated, ranging from 3 (lowest level) to 18 (highest level).

#### Obesity‐Related Problems Scale

The Obesity‐related Problems (OP) Scale is an obesity‐specific QoL instrument developed for measuring the impact of obesity on psychosocial functioning in 2 domains: distress and avoidance [18-20]. Participants are asked to rate how bothered they are by their body size in different social situations and to what extent they avoid such situations. Scale scores range from 0 to 100, and higher scores indicate more dysfunction. A distress score of <40 is interpreted as mild, 40‐59 as moderate, and ≥60 as severe dysfunction.

#### Experiences of Treatment Effects

Study-specific questions about the participants’ experiences of the treatment effects were used at the 12-month follow-up. We asked whether the treatment had helped them in changing their lifestyle habits and whether the treatment had had positive effects on their health and well-being. A 5-point response scale was used for perceived changes in lifestyle habits (Not at all, A little, Pretty much, A lot, and Very much) and for perceived treatment effects (No positive effects, Small positive effects, Fairly large positive effects, Great positive effects, and Very large positive effects).

#### Treatment Satisfaction

After the participants had completed each treatment module, they were asked to rate how satisfied they were with the content and how relevant or meaningful the practice task was. The questions were answered on a 5-point response scale with the anchors 1 (Poor) to 5 (Very good) for treatment satisfaction and the anchors 1 (Not relevant) to 5 (Very relevant) for satisfaction with the practice tasks.

### Interview Procedure for Dropouts

Participants who dropped out of the treatment were invited to participate in a telephone interview about their experiences with the treatment and their reason for dropping out. Prior to the interviews, the participants received oral information about the aim of the interviews. A semistructured interview guide was used, covering areas such as previous experience of digital working methods; experience of the modules of the IDT-O; amount of time needed to complete the modules, including the practice tasks; experiences of their digital contact with the therapist; and whether they had experienced any effects of their participation in the IDT-O. All interviews started with a question about their reason for dropping out. The questions were based on both clinical and scientific interest in the further development of the IDT-O.

The semistructured interviews were conducted by a researcher with nursing background experienced in qualitative methods (first author [AAC]), who had no previous relationship with the participants or the staff at the obesity unit. The duration of the interviews varied within 18‐69 minutes (mean 31, median 24.5 minutes). Interviews were digitally recorded, after consent from the informants, and were transcribed verbatim by a professional transcriptionist. There was a variation in time span from patients dropping out from the intervention and being interviewed. This was partly because it was not always explicit when a participant had decided to discontinue the intervention. Sometimes they just stopped fulfilling their tasks and did not reply to the therapists’ messages. The longest interval between dropping out and being interviewed was therefore just less than 4 months.

### Statistical Analysis

The mean and SD are presented for continuous variables, and relative frequencies are provided for categorical variables. The effect size (ES) of a change between baseline and 6- and 12-month follow-up was estimated by calculating the standardized response mean (SRM)—that is, the mean change divided by the SD of change. ES or SRM was evaluated according to standard criteria: <0.20=trivial, 0.20‐0.49=small, 0.50‐0.79=moderate, and ≥0.80=large [21]. The sample size was too small for significance testing of the treatment outcomes. Analysis was performed using SAS (version 9.4; SAS Institute Inc).

### Qualitative Inductive Content Analysis

The analysis was inspired by the steps described by Graneheim and Lundman [22]. First, the first author (AAC), who both conducted the interviews and is experienced in qualitative methods, listened to all the interviews, corrected any errors from the transcriptions, and attempted to get a sense of the whole. Next, the interviews were imported into NVivo software (QSR International) for further analysis. Meaning units were identified and labeled with a code illustrating the content. Thereafter, the codes were scrutinized for similarities and differences and were clustered into categories describing the manifest content of the interview data. Finally, the categories were reflected on, and their latent content was identified in the form of subthemes and a main theme. The analysis was performed by the first author (AAC) and, in order to ensure rigor and trustworthiness, was later discussed with the other authors. The other authors all had experience of working with patients living with obesity, working as a licensed psychologist (JK), a general practitioner (SJ and MG), or a public health nurse (AI). During the process of analysis, the authors practiced reflexivity by discussions comparing their preunderstanding with what was revealed in the dataset [23].

### Ethical Considerations

This study was approved by the Swedish Ethical Review Authority (registration number 2019‐04695). At the first visit to the obesity unit, participants were informed orally and in writing about the study, and written consent was obtained. No compensation was offered to the participants, but the intervention and the health care visits included in the study were offered free of charge. The quantitative and qualitative data were deidentified before analysis. When presenting quotes in the “Results” section, this was done cautiously, ensuring that the information could not be traced back to a certain participant. All quotations have been marked with the participation number, instead of names.

## Results

### Overview

A total of 20 participants started the IDT-O program and 35% (7/20) completed all 12 modules. Of the participants, 17 were females, the mean age was 44.2 (SD 16.4) years, and about half (11/20, 55%) had a university education (Table 2). Mean body weight was 117.1 (SD 19.8) kg, and mean BMI was 42.8 (SD 6.4) kg/m^2^. Ten (8 females) out of 13 dropouts agreed to participate in an interview. Two participants could not be reached, and 1 declined participation. The majority of the participants had previous experience with weight-reducing interventions. There was variation in the number of modules participants completed before dropping out.

**Table 2.** Baseline characteristics of the 20 participants who started treatment, the 7 participants who completed the 6-month treatment, and those who dropped out and participated in the interview study.

Characteristics	Started treatment	Completed treatment	Dropouts interviewed
Participants, n (%)	20 (100)	7 (35)	10 (50)
Sex, n (%)			
Males	3 (15)	0 (0)	2 (20)
Females	17 (85)	7 (100)	8 (80)
Age (years)			
Age, mean (SD)	44.2 (16.4)	49.4 (18.3)	42.9 (15.8)
Age range	20‐77	27‐77	26‐73
Age groups, n (%)			
20‐34	6 (30)	2 (28.6)	3 (30)
35‐49	8 (40)	2 (28.6)	4 (40)
50+	6 (30)	3 (42.9)	3 (30)
Education, n (%)			
Mandatory	1 (5)	0	0
High school	6 (30)	2 (28.6)	3 (30)
University	11 (55)	5 (71.4)	6 (60)
Other	2 (10)	0	1 (10)
Body weight (kg)			
Body weight, mean (SD)	117.1 (19.8)	111.2 (18.0)	123.0 (20.0)
Body weight range	84.6‐159.0	84.6‐137.5	89.7‐159.0
BMI (kg/m^2^)			
BMI, mean (SD)	42.8 (6.4)	43.1 (7.1)	43.6 (6.6)
BMI range	33.5‐57.7	33.5‐51.4	35.9‐57.7
BMI interval, n (%)			
28‐29	0	0	0
30‐34.9	1 (5)	1 (14.3)	0
35‐39.9	7 (35)	2 (28.6)	3 (30)
40‐44.9	5 (25)	1 (14.3)	3 (30)
45‐49.9	4 (20)	1 (14.3)	3 (30)
50+	3 (15)	2 (28.6)	1 (10)

### Outcome Measures

Of those who completed the 6-month treatment, the mean percent weight reduction was 2.1% (95% CI 0.1‐4.0) after 6 months and 2.0% (95% CI −1.1 to 5.1) after 12 months (Table 3). Two participants achieved a weight reduction of at least 5% after 12 months.

**Table 3.** Treatment outcomes at 6- and 12-month follow-up for the 7 participants who completed the 6-month treatment program.

Outcome measures	Baseline	6-month follow-up	12-month follow-up
Body weight (kg)			
Mean (SD)	111.2 (18.0)	109.0 (18.5)	109.0 (18.5)
Change, mean (95% CI)	—^a^	−2.2 (−4.2 to 0.1)	−2.2 (−5.8 to 1.4)
Change (%), mean (95% CI)	—	−2.1 (0.1 to 4.0)	−2.0 (−1.1 to 5.1)
BMI (kg/m^2^)			
Mean (SD)	43.1 (7.1)	42.3 (7.3)	42.2 (6.9)
Change, mean (95% CI)	—	−0.8 (−1.7 to 0.1)	−0.9 (−2.3 to 0.6)
Eating habits^b^			
Mean (SD)	6.1 (3.3)	7.3 (2.2)	6.9 (1.7)
Change, mean (SD)	—	1.1 (2.3)	0.7 (2.8)
SRM^c^ (95% CI)	—	0.50 (−0.31 to 1.28)	0.25 (−0.51 to 1.00)
Physical activity^d^			
Mean (SD)	8.1 (2.9)	11.4 (4.6)	12.7 (3.9)
Change, mean (SD)	—	3.3 (5.9)	5.0 (5.4)
SRM (95% CI)	—	0.56 (−0.26 to 1.35)	0.93 (−0.08 to 1.87)
OP^e^			
*Distress,* mean (SD)	52.4 (25.5)	55.0 (29.2)	41.8 (21.7)
Change, mean (SD)	—	2.6 (14.7)	−10.6 (24.8)
SRM (95% CI)	—	−0.17 (−0.92 to 0.58)	0.43 (−0.37 to 1.19)
*Avoidance,* mean (SD)	34.1 (20.1)	24.9 (16.6)	22.7 (14.7)
Change, mean (SD)	—	−9.2 (10.4)	−11.4 (16.9)
SRM (95% CI)	—	0.89 (−0.03 to 1.75)	0.67 (−0.18 to 1.48)

a Not available.

b The score ranges from 0 to 12, with a higher score indicating more favorable dietary habits.

c SRM: standardized response mean (effect size of change). SRM criteria: <0.20 = trivial; 0.20‐0.49 = small; 0.50‐0.79 = moderate; and ≥0.80 = large.

d Self-reported physical activity on a scale from 3 to 18, where a higher value indicates more minutes per week spent on physical activity.

e OP: Obesity‐related Problems scale (score range 0‐100). Higher distress scores indicate more obesity-related psychosocial distress in social situations, and higher avoidance scores indicate more avoidance of social situations.

In the completers, dietary habits scores indicated a moderate improvement (SRM 0.50, 95% CI −0.31 to 1.28) after 6 months and a small improvement (SRM 0.25, 95% CI −0.51 to 1.00) after 12 months (Table 3). The change in self-reported physical activity showed a moderate improvement (SRM 0.56, 95% CI −0.26 to 1.35) after 6 months and a large improvement (SRM 0.93, 95% CI −0.08 to 1.87) after 12 months.

No change in OP distress scores was noted after 6 months (SRM 0.17, 95% CI −0.92 to 0.58), while a small improvement (SRM 0.43, 95% CI −0.37 to 1.19) was observed after 12 months. The change in OP avoidance scores indicated a large improvement (SRM 0.89, 95% CI −0.03 to 1.75) after 6 months and a moderate improvement (SRM 0.67, 95% CI −0.18 to 1.48) after 12 months.

The responses at the 12-month follow-up to the question of whether the treatment had helped in changing participants’ lifestyle habits were as follows: 42.9% (3/7) “a little,” 28.6% (2/7) “pretty much,” 14.3% (1/7) “a lot,” and 14.3% (1/7) “very much” (Table S1 in Multimedia Appendix 1). The answers to whether the treatment had positive effects on participants’ health were as follows: 28.6% (2/7) “small effects,” 42.9% (3/7) “fairly large effects,” and 28.6% (2/7) “great effects.” The same response pattern was obtained for the question about positive effects on well-being.

In completers, the mean treatment satisfaction rating for the 12 modules was 4.5 (range 3.7‐4.9, where 1 = Poor and 5 = Very good), while the mean rating for the relevance or meaningfulness of the practice tasks was 4.6 (range 4.0‐5.0, where 1 = Not relevant and 5 = Very relevant) (Table 4). The dropouts’ mean satisfaction ratings for the completed modules and the practice tasks were about the same as those of the completers. Dropouts completed on average 4.4 modules with a range of 1-9 modules.

**Table 4.** Satisfaction ratings with the 12 treatment modules and the practice tasks for those who completed the 6-month treatment program and for dropouts.

Treatment module	Satisfaction with treatment^a^		Satisfaction with practice task^b^	
Completers (n=7), mean	Dropouts (n=13), mean	Completers (n=7), mean	Dropouts (n=13), mean
1	3.7	4.4 (n=12)	4.0	4.5 (n=12)
2	4.0	4.6 (n=13)	4.6	4.5 (n=13)
3	4.9	4.5 (n=11)	5.0	4.5 (n=11)
4	4.2	4.0 (n=5)	4.5	4.6 (n=5)
5	4.7	5.0 (n=3)	4.6	5.0 (n=3)
6	4.9	4.0 (n=1)	4.7	5 (n=1)
7	4.6	4.0 (n=1)	5.0	4.0 (n=1)
8	4.7	5.0 (n=2)	4.9	5.0 (n=2)
9	4.6	4.0 (n=1)	4.6	5.0 (n=1)
10	4.6	—^c^	4.7	—
11	4.3	—	4.4	—
12	4.2	—	4.3	—
Mean 1‐12	4.5	—	4.6	—

a The content of the treatment module has generally been ___ (answered using a 5-point response scale with anchors from 1 [Poor] to 5 [Very good]).

b Was the practice task for the module relevant/meaningful? (answered using a 5-point response scale with anchors from 1 [Not relevant] to 5 [Very relevant]).

c Not available.

### Qualitative Findings

The analysis resulted in 1 main theme—namely, *The programme was OK, but it does not suit everyone*—which was the latent content of all the data running through the categories and subthemes. Despite having dropped out from the IDT-O, the participants mainly believed that the content was relevant and of good quality; nevertheless, it did not suit them—at least not at this specific time in life. The 3 subthemes focus on 3 main messages. The first of these, *It wasn’t for me,* highlights issues explaining why the IDT-O did not suit some participants at this stage of life. The second subtheme, *There were good things*, points to aspects that were found to be valuable. The third subtheme, *There are things to improve*, comprises ideas that are worth considering in order to further improve the IDT-O. Table 5 provides an overview of the results.

**Table 5.** Overview of the qualitative findings: main theme, subthemes, and categories.

Main theme	“The programme was OK, but it does not suit everyone”		
Subthemes	It wasn’t for me	There were good things	There are things to improve
Categories	The treatment wasn’t in line with my expectations and needs. It wasn’t the right timing for me to participate. There were some aggravating factors in my everyday life complicating participation.	The content is relevant for people like me. Internet treatment offers freedom. The therapist was supportive.	Parts of the programme and the digital platform were not user-friendly. The supportive aspect should be further developed.

#### It Wasn’t for Me

The subtheme *It wasn’t for me* is built on the categories *The treatment wasn’t in line with my expectations and needs*, *It wasn’t the right timing for me to participate* and *There were some aggravating factors in my everyday life complicating participation*.

##### The Treatment Wasn’t in Line With My Expectations and Needs

There were various reasons why participants believed that the IDT-O was not the right treatment option for them, having to do with the content or mode of delivery. As many participants had been living withobesity for a long time and had tried to lose weight before, they experienced the content as being too basic and adding nothing new. Furthermore, it did not match their expectations. They claimed that treatment needed to be individually adapted, and some said that they had expected to be offered more specific advice regarding what to eat, instead of treatment influenced by CBT. Some were uncertain about the explicit goals of the treatment, while they themselves had joined the IDT-O to lose weight. One participant said, “Well, the introduction, or whatever you call it, was very informative. They had put together information about overweight in a good way, but what I miss is the help to deal with overweight, that’s what it lacks” (Participant ID 1).

There were also accounts of dropping out of treatment because of self-blame during modules focusing on recording the participants’ own behaviors. The participants already knew that their behaviors were not ideal and did not want to repeatedly consider that. Instead, they wanted support focusing on how to change these behaviors, as well as getting tips and exercises tailored to their needs.

With regard to the mode of delivery, some felt alone in the digital treatment and wished for at least 1 in-person visit with the therapist. Half of the participants also reported difficulties finishing the treatment modules in time, especially when the practice task involved reflecting on a specific topic. Therefore, the participants suggested that they themselves should be able to decide their pace of working through the modules, as it was difficult to catch up when they fell behind. For example, a participant commented, “... So, I ended up behind, and then I found it overwhelming to catch up” (Participant ID 5).

##### It Wasn’t the Right Timing for Me to Participate

A common explanation for dropping out from the IDT-O was that the timing was not right for participation. Many of the dropouts revealed that their life situation was turbulent. For example, some had started a new job, were just about to separate, or were moving. Another reason was illness, either their own or a family member’s: ‘Well, I believe I could have managed to fulfil my engagement with the programme if it hadn’t been for Covid and if things had been more settled around me. However, this simply wasn’t the case” (Participant ID 10). There were also accounts of realizing that they needed to deal with their own existing psychological health issues before starting treatment to lose weight. Another participant specifically mentioned that her poor walking ability influenced her decision to drop out of the IDT-O, as she found it impossible to complete the modules that focused on physical activities.

##### There Were Some Aggravating Factors in My Everyday Life Complicating Participation

Some participants reported that, despite the freedom to attend the IDT-O from a place of their choice, they had experienced unexpected difficulty regarding finding a time and place to sit and concentrate on reading the modules and doing the practice tasks. One participant noted, “Being a parent of young children, you simply don’t have the time” (Participant ID 3). Thus, some of the participants postponed their tasks and did not finalize their assignments until they received a reminder from the treatment platform or therapist.

### There Were Good Things

The subtheme *There were good things* focuses on the participants’ positive comments about the IDT-O, comprising the categories *The content is relevant for people like me*, *Internet treatment offers freedom*, and *The therapist was supportive*.

#### The Content Is Relevant for People Like Me

The participants regarded the content of the IDT-O as relevant for people with obesity, and they could theoretically see themselves participating in the IDT-O on another occasion. Although some of the content was not new to them, they still found it worth repeating. Participants specifically identified the module focusing on recording their food intake as beneficial, as it created a base for reflecting on their behavior in relation to eating. Although it was not always easy to adopt the theory in practice, participants reported that they had changed some behaviors in their daily life, despite dropping out from the IDT-O:

*Actually, I’ve changed. I am better at having breakfast after this programme. Because, before, I hardly ever had breakfast...And, after this programme, I’ve started to have some breakfast—well, perhaps not every day, but on four mornings out of seven. Previously, I had breakfast 1 out of 7 days at the most (laughs). Yes, so that’s something I’ve changed; yes, I more frequently have breakfast (laughs)*.

(Participant ID 4)

Other examples of changed behavior included reflecting more on what to eat and how to think about food intake and physical activity, and making more conscious choices, such as adjusting portion sizes. The content about obesity being defined as a chronic illness made one person feel less guilty about the condition, while another participant questioned the relevance of such knowledge.

#### Internet Treatment Offers Freedom

One major benefit of participating in the IDT-O was the participants’ freedom to read or watch the content in the modules or to work with practice tasks at a time most convenient to them. Some worked at odd hours, and some had small children who needed attendance. In such cases, IDT was feasible: “It worked really well for me, having access to it [the programme] whenever convenient, but, as I said, when having children and working full-time, you have to squeeze it into your life whenever possible” (Participant ID 9). Some explained that, for them, it would not have been possible to attend on-site meetings at scheduled times. The IDT made it possible for them to work at a pace that suited them; moreover, if they were ill, they did not necessarily miss a treatment visit. In addition, the participants found it practical to work from home.

#### The Therapist Was Supportive

The therapists acted in a way that made the participants feel understood and respected; they were perceived as encouraging, although some participants described a need for more constructive criticism: “Well,...ehm...but they have been encouraging as well, but they haven’t.... you need to hear bad things as well; well, it has to be both for it to work” (Participant ID 7). The therapists offered advice and took time to comment on the practice tasks, although some participants perceived these comments to be too general. When the participants were late completing their practice tasks, the therapists repeatedly sent reminders and tried to motivate the participants to stay with the IDT-O. These reminders were regarded as helpful.

### There Are Things to Improve

Although the participants were positive to a certain degree, they also had insights about possible drawbacks. The categories in this subtheme are *Parts of the programme and the digital platform were not user-friendly* and *The supportive aspect should be further developed*.

#### Parts of the Programme and the Digital Platform Were Not User-Friendly

One major issue with regard to non–user-friendliness was the amount of text participants had to go through. The bulky text could be experienced as overwhelming, making it difficult to get an overview and comprehend the content. For some, just the sight of the amount of text had made them close the program and postpone their reading to another day.

The amount of text also caused problems for some working via a mobile phone; it was difficult to read the text in small font and in a nonpage format, where the text just continued. Furthermore, it was inconvenient to report extensive practice tasks using a mobile phone. However, it was acceptable to use a phone for shorter communication with the therapist.

As some of the participants had problems working on a computer due to concentration issues, they would have preferred to print out the modules and practice tasks. However, it was difficult to figure out how to print the text, and the platform did not permit printing more than 1 page at the time (ie, instead of a whole chapter).

Participants with dyslexia said that they would have benefitted from an audio function to be able to listen to the content of the IDT-O instead of reading it. When they worked on their own, the practice task took them a considerable amount of time, making it necessary for a family member or a friend to assist with some of the reading. Although these participants informed the health care professionals about their reading problems when they were first included in the IDT-O program, they were advised to try it anyway: “...Yes, they said I could give it a try, despite my problems, so... [I did]” (Participant ID 2).

The digital platform for the IDT-O was regarded as not very user-friendly. There were multiple pages, and many clicks were needed to reach the IDT-O and the content of a module, to submit practice tasks and to read comments from the therapist. This hindered some participants, who would have liked to make notes and comments while on the move. One participant had problems with saving the answers to assignments, which was frustrating and time-consuming, as the information needed to be entered again.

#### The Supportive Aspect Should Be Further Developed

As feedback was an important aspect of the treatment, participants claimed that they would have liked to meet with the therapist at least once during the treatment period, or for follow-up to have been offered by phone. They believed that a meeting would have offered a more personal dimension to their treatment. Another major drawback was the insufficient individualized support and advice; also, no one actually followed up on whether the participants adhered to their own plan or what the results of certain activities were:

*What works best for me, that’s when showing up at the therapist and being asked, “How have things worked out for you?” I sort of need to have someone to report to, someone somewhat keeping an eye on me, keeping control.*

(Participant ID 8)

General encouragement was not regarded as facilitating enough in relation to the needs of the participants. Some also mentioned that they would have benefited more from a group intervention.

## Discussion

### Principal Findings

This pilot study was a first step in evaluating the feasibility and effectiveness of an IDT program for individuals with obesity. Both qualitative and quantitative methods were used to assess the intervention. The findings indicate that the IDT-O holds potential as a treatment option for people with obesity, although only 35% (7/20) of the participants completed the program. The quantitative and qualitative findings show that the participants were generally satisfied with the content and design of the intervention. Those who completed the IDT-O lost some weight, reported improved dietary habits, increased physical activity, and improved psychosocial functioning, and maintained these behaviors for 6 months after completing the treatment. However, the quantitative findings were based on only 7 participants who completed the IDT-O and should be considered preliminary. The qualitative findings, defined in the 3 subthemes: “It wasn’t for me,” “There were good things,” and “There are things to improve,” also revealed that the intervention was not deemed useful by everyone, and various reasons for dropping out were provided. The interviewees also offered suggestions that may be useful for program improvements.

The efficacy of a digital intervention is linked to user motivation and engagement, with nonadherence being a common problem [24]. Studies show that almost half of those who start iCBT treatment do not complete the program [25,26]. In this study, 65% (13/20) of the participants dropped out of treatment, typically early in the program, indicating that the IDT-O program did not suit all participants. Some participants had unmet expectations for the treatment; for example, they wanted more detailed advice, more contact with the therapist, or had difficulty concentrating and reading. Some dropouts were due to factors external to the treatment, such as unforeseen circumstances in life that affected participation. This study was conducted during the Covid-19 pandemic, which may have contributed to the high dropout rate due to participants’ own or family members’ illness. Aside from this factor, the reasons for dropout identified in this study are broadly consistent with the findings of a qualitative study on nonadherence to iCBT for generalized anxiety disorder [24], as well as with patients’ and therapists’ experiences of challenges related to other internet-delivered interventions [9,27,28]. Given the issue of nonadherence, this finding underscores the importance of thoroughly assessing patients’ expectations and clarifying the goal of the program before offering such treatment [4], although doing so can be challenging [9]. Such assessments are crucial, both from a professional standpoint and for the patients, as both parties invest resources in the treatment. Additionally, patients who do not complete the treatment may experience feelings of guilt and shame, as the treatment is largely based on their own initiative [29,30].

Both the dropouts and the completers reported high satisfaction ratings regarding the completed modules and practice tasks, although this comparison was limited to the initial 5 out of 12 program modules. This positive view of the program content was supported by the findings based on interviews with the dropouts. The completers demonstrated improvements in self-reported dietary habits, physical activity, and psychosocial functioning at follow-up, in line with previous research [13]. The completers also stated that the IDT-O program had helped them change lifestyle habits and had positive effects on health and well-being. However, due to the limited number of participants who completed the program, the observed changes are uncertain and must be confirmed in a more comprehensive study. Interestingly, the dropouts also reported that they had benefitted from the content of the modules they had completed and had made some changes to their dietary habits.

The weight loss among completers 12 months after starting treatment was modest (–2.2 kg) and slightly lower than that observed in previous studies. In a comprehensive review including 44 studies on web-based obesity interventions, the average weight loss was –3.5 kg (range –0.1 to –7.8 kg) after treatments lasting between 2 and 24 months [12]. The modest weight loss might be explained by the fact that the content of the IDT-O program is not exclusively focusing on weight loss, but on change of behaviors, which is not a quick fix. From both a public health and individual perspective, even moderate weight loss is considered relevant [31,32], and improvements in dietary habits and increased physical activity after treatment can have further long-term positive effects on weight and health [33]. To increase the effectiveness of weight management, integrating the IDT-O program with pharmacological treatment for weight loss may facilitate the achievement of more substantial long-term weight reduction and weight maintenance thereafter. In a recent publication, the American Diabetes Association recommended that pharmacological obesity treatment should be used in combination with other therapies, focusing on nutrition, physical activity, and behavioral changes, to achieve health goals. They base their recommendations on research showing that addition of pharmacological treatment to lifestyle interventions improves weight reduction and weight loss maintenance [34].

Obesity is a stigmatized condition [33], and reducing weight stigma was one of the goals of the IDT-O program. In the study, the OP scale was used to measure stigma-related impacts of obesity on psychosocial functioning, a key domain in the assessment of QoL in people with obesity. Among the completers, improvements in psychosocial functioning (distress and avoidance) were noted at follow-up, likely due to a combined effect of weight loss and behavioral treatment. Reducing body weight and weight stigma can be complementary goals in obesity treatment. By focusing on reducing internalized stigma, beneficial effects on general health and QoL may be achieved, independent of weight reduction [33,35].

In this intervention, only written online communication between the therapist and the participant was used. The interviewed participants expressed a need for increased contact with the therapist and suggested that face-to-face or telephone contact should be included during the treatment—a finding consistent with other qualitative studies of iCBT [24,28,36]. Blended treatment, which combines online communication with face-to-face meetings, has been proposed as a means of enhancing iCBT interventions [7]. The use of video or phone consultations as supplements to written online communication is another option. However, the potential benefits of blended treatment are largely unknown, and further research is needed to determine the most effective mix of online and face-to-face consultations and whether or not the therapeutic alliance is increased by such modifications. Moreover, the potential benefits must be weighed against the increased costs of a blended intervention.

Some participants found it difficult to adhere to the standardized 2-week schedule for each module and suggested that the treatment should be more flexible and tailored to individual needs. This is a common reaction to internet-delivered therapy [24,28,37]. There were many reasons given for not managing the time limit of the scheduled modules. One that might need extra consideration was that the amount of text could be overwhelming for those with reading difficulties or having problems concentrating. The notion of extensive text to read [27] and the requirement for reading and writing skills [24] call for future innovative and assessable solutions, including text-to-speech features.

Due to the incomplete adaptation to mobile devices, participants encountered difficulties accessing the program via their phones. Given that mobile phones are the most common gateway to the internet today [38], it is critical for IDT programs to be fully optimized for mobile devices. As in the current findings, technical issues are a recurring topic when investigating patients’ experiences with IDTs [4,9,29]. These issues should be addressed when offering these treatments to patients or when developing IDTs, as technical issues have been given as a reason for dropping out of treatment [27].

### Strengths and Limitations

The IDT-O program was evaluated in a real-life clinical setting without extra resources, which is a strength with regard to its further development and future implementation. However, the therapists were inexperienced with IDT, which may have affected the results of this study.

The use of qualitative and quantitative methods, along with the inclusion of data from both completers and dropouts, resulted in comprehensive data, which allowed us to evaluate different perspectives. The findings of this pilot study are valuable for designing a full-scale study and for further refining the IDT-O program. The trustworthiness of this study was enhanced by the fact that the interviewer was a neutral party, uninvolved in the design of the IDT-O program and with no prior or ongoing therapeutic relationship with the interviewees. In addition, the mix of the researcher’s professional backgrounds and preunderstanding contributed to the credibility.

Despite the strengths of the study, there are also some limitations to consider. First, the sample size was too small for significance testing; instead, we calculated effect sizes (ESs) to describe changes over time for those who completed the IDT-O. However, the ES estimates are uncertain because they are based on a small number of participants. Second, the dropout rate was higher than expected, but most of the dropouts participated in the qualitative interviews and shared their experiences, adding valuable insights to the evaluation of the IDT-O program. Third, the Covid-19 restrictions may have affected participants’ daily life habits regarding physical activity and eating habits [39], which in turn may have influenced the outcomes of this study.

### Conclusions

In many respects, the IDT-O can be deemed feasible as a treatment option for people with obesity. Among those who completed the IDT-O, body weight decreased and dietary habits, self-rated physical activity, and psychosocial functioning improved. Despite these positive findings, the dropout rate was high, with participants indicating different reasons for discontinuing the treatment. Therefore, more research is needed before concluding the positive results. The results can, together with existing literature on the topic, form a base for future development and further evaluation of the IDT-O. Suggestions for further development of the IDT-O include adding a text-to-speech feature, improving the platform’s compatibility with mobile phones, and enhancing its overall accessibility and usability. In the future, we recommend a more tailored assessment of potential patients before offering the IDT-O, further development of the technical aspects of the platform, an evaluation of the benefits of adding individual visits with the therapist, and testing of the program when combined with other treatment regimens (eg, pharmacotherapy) for obesity. These modifications could potentially lead to greater effects on various aspects of patients’ health.

## Supplementary material

10.2196/79853 Multimedia Appendix 1 Experiences of treatment effects after 12 months in the 7 participants who completed the treatment program.

10.2196/79853 Checklist 1 COREQ (Criteria for Reporting Qualitative research) checklist.

## Abbreviations

CBT: cognitive behavioral therapy
ES: effect size
iCBT: cognitive behavioral therapy via the internet
IDT: internet-delivered treatment
IDT-O: internet-delivered program for obesity
OP: Obesity-related Problems Scale
QoL: quality of life
SMART: Specific, Measurable, Achievable, Relevant, and Time-bound
SRM: standardized response mean
