# Supplementary material for: Experiences From an Internet-Delivered Treatment Program for Individuals With Obesity: Pilot Study
Source: JMIR Form Res. 2026 Apr 17;10:e79853. doi: 10.2196/79853 (PMC13089626; doi:10.2196/79853)
Supplement: Multimedia Appendix 1 [file formative-v10-e79853-s001.docx]

**Multimedia Appendix 1**

Table S1. Experiences of treatment effects after 12 months in the 7 participants who completed the treatment program.

| **Questions** | **Response alternatives** | | | | |
| --- | --- | --- | --- | --- | --- |
|  | *Not at all*  n (%) | *A little*  n (%) | *Pretty much*  n (%) | *A lot*  n (%) | *Very much*  n (%) |
| Do you think the treatment has helped you change your lifestyle? | 0 | 3 (42.9) | 2 (28.6) | 1 (14.3) | 1 (14.3) |
|  | *No positive effects*  n (%) | *Small positive effects*  n (%) | *Fairly large positive effects*  n (%) | *Great positive effects*  n (%) | *Very large positive effects*  n (%) |
| Do you think the treatment has had positive effects on your health? | 0 | 2 (28.6) | 3 (42.9) | 2 (28.6) | 0 |
| Do you think the treatment has had positive effects on your well-being? | 0 | 2 (28.6) | 3 (42.9) | 2 (28.6) | 0 |
